# What Variables Differentiate between Selected and Not Selected High-Performance Adolescent Track and Field Athletes?

**DOI:** 10.5114/jhk/193995

**Published:** 2024-12-19

**Authors:** Antonio E. Vélez-Alcázar, Juan Alfonso García-Roca, Raquel Vaquero-Cristóbal

**Affiliations:** 1 Faculty of Sport Sciences, Universidad Católica de Murcia (UCAM), Murcia, Spain.; 2 Centre for Olympic Studies, Universidad Católica de Murcia (UCAM), Cartagena, Spain.; 3 Research Group Movement Sciences and Sport (MS&SPORT), Department of Physical Activity and Sport, Faculty of Sport Sciences, University of Murcia, Murcia, Spain.

**Keywords:** body composition, youth, physical fitness, performance, maturity

## Abstract

Early detection of young talent and athlete development programs lack reliable and valid indicators that can predict future success. Therefore, the aim of the present study was to analyse the differences between selected and unselected athletes to define the determinant factors, as well as the influence of gender on the variables that could predict athletic success. The research was carried out using a cross-correlational descriptive design involving 95 athletes, of whom 46 were males (average age = 18.31 ± 2.31 years) and 49 were females (averaged age = 17.27 ± 1.44 years), and whose sociodemographic, psychological, physical and anthropometric variables were analysed. Significant differences were observed in maturity (p < 0.001), anthropometric variables related to bone structure, muscle mass, and body mass (p < 0.001 to 0.044), physical condition variations related to strength, power, sprint ability, flexibility, and balance (p < 0.001 to 0.013), as well as athletic experience and training variants (p = 0.003–0.004). These results should be taken into account for the sporting programming of young athletes and in order to be aware of the modulating effect of biological maturity and its influence on athletic performance.

## Introduction

Numerous studies have highlighted the benefits of a proper selection process of young sports talent for clubs. These include early specialization in sport skills, the opportunity to integrate young athletes into high-level teams, and long-term financial security (Peña-González et al., 2018). However, making poor decisions in the sport talent selection process has serious economic repercussions for sports clubs (Johnston and Baker, 2020), and can result in “waste of talent”, a concept that refers to the high number of talented athletes who might be excluded from competitive sport opportunities due to poor decisions during this process (Pinder et al., 2013).

Despite the proliferation of early talent detection and athlete development programs in sport, most of them lack reliable and valid indicators that can accurately predict success in sport performance in adulthood (Baker et al., 2018). Talent detection programs often operate under the assumption that talent observed at early ages will follow a predictable trajectory in future sports performance (Johnston and Baker, 2020). However, this assertion has been refuted by scientific research, which points to the multidimensionality of factors that may explain why an athlete becomes a high-level athlete in adulthood (Gonçalves et al., 2012; Krzysztofik et al., 2024).

In response to this, in recent years, more accurate methodologies and models have been developed to predict sport talent, which consider the multidimensionality of performance (Peña-González et al., 2018). Predictor variables include physical condition, anthropometric characteristics, as well as growth and maturation (Albaladejo- Saura et al., 2021), among others. More specifically, physical and physiological changes during biological maturation occur at different rates in each individual (Malina and Bouchard, 1991), so that competitions organised according to chronological age present discrepancies in the maturation status of athletes. Thus, early maturers tend to have a competitive advantage during the growth stage in sports that require power, strength, speed, and agility (Albaladejo-Saura et al., 2021), as a result of their physiological and anthropometric differences (Albaladejo-Saura et al., 2021; Gonçalves et al., 2012). However, these differences disappear once the growth period ends and adulthood is reached, meaning that the advantage based on these characteristics is temporary (Gonçalves et al., 2012). Therefore, basing sport talent selection processes solely on physical fitness and anthropometric variables, without considering the effect of maturation on these characteristics, renders these markers of little use in selection strategies, and increases the chances of “waste of talent” (Pearson et al., 2006).

Track and field is no stranger to this phenomenon. It is a sport discipline in which individual and club competitions are held, as well as competitions for national and territorial selections of the most outstanding athletes (Real Federación Española de Atletismo, 2024). For individual competitions, the International and National Athletics Federation differentiates between athletes, for both competition and classification, according to sex into male and female, from U-14 up to and including the senior level (Real Federación Española de Atletismo, 2024). Regarding club competitions, these are also organized according to sex, separating males from females from the U-14 and up to the senior category (Real Federación Española de Atletismo, 2024). As for the selection process for the inclusion of male and female athletes in youth talent development programs or competitions, it is usually carried out by scouts or coaches based on the observation of athletes during training and competitions (Ruud et al., 2018) or the athlete’s performance in individual and club competitions (Federación de Atletismo de la Región de Murcia, 2023; Real Federación Española de Atletismo, 2023). Therefore, the decision to include or exclude male and female athletes in programs or competitions is mainly based on general impressions (Krogh Christensen, 2009), which may be biased (Ruud et al., 2018), and variables such as sporting performance, which may be influenced at the growth stage by the state of maturation (Albaladejo-Saura et al., 2021). As a consequence of the above, these processes tend to exclude late matures due to their lower performance as compared to their peers during the formative stages (De Subijana and Lorenzo, 2018).

The existing literature shows a lack of consensus on the variables that coaches and scouts should consider in order to establish objective selection criteria in the detection of talent in athletics. Furthermore, no studies have been carried out on the influence of sex in this process, despite the fact that in other sports, it has been found that sex can be a determining factor in the variables that impact the selection of athletes (Albaladejo-Saura et al., 2023). This could be due to the existing biological differences (characteristics of reproductive structure, functions, phenotype, and genotype) that differentiate the male from the female organism, following the definition of “sex” from the National Library of Medicine of the United States government (National Library of Medicine of the United States government, 2024). At this point, it is important to differentiate the concept of “sex”, which focuses on biological differences, from “gender identity”, defined by the same institution as “a person's self-concept as male and masculine or female and feminine, or ambivalent, based in part on physical characteristics, parental responses, and psychological and social pressures. It is the internal experience of the gender role” (US Government National Library of Medicine, 2024). Previous studies have shown that during puberty, a series of biological changes occur that result in the appearance of sexual dimorphism that could condition sports performance of athletes during puberty and beyond (Albaladejo-Saura et al., 2021). In addition, maturational changes in males and females do not occur at the same rate (Malina and Bouchard, 1991). It is therefore necessary to analyse the influence of sex on the phenomenon of sports talent detection in athletics.

Therefore, the objectives of the present research were: a) to analyse the differences between the selected and non-selected athletes of the technification programmes from the territorial selections, with respect to sociodemographic, training, psychological, physical condition, anthropometric and derived variables of athletes in training; and b) to analyse the impact of sex on the differences between the selected and non-selected athletes of the technification programmes from the territorial selections in the aforementioned variables. The hypotheses were that: a) maturation, physical condition and anthropometric variables affected by it would be the main differentiating factors between selected and non-selected athletes in the technification programmes of the territorial selections; and that b) sex as a covariate could influence the determining variables for an athlete to be selected or not.

## Methods

### Study Design

A cross-sectional descriptive-correlational design was carried out. We compared the data from a group of athletes selected by the Athletics Federation of Murcia (FAMU) for participating in the sports technification programmes organised by that federation, with a group composed of high-level athletes of the same age competing for club teams in the national league category, who had not been included in the FAMU sports technification programme. The differences between these groups were determined in terms of sociodemographic variables, sports, biological maturity, anthropometry and physical condition. The data were collected in November 2022.

Prior to beginning the research, the Ethics Committee of the Catholic University of Murcia approved the research study protocol (protocol code: 052303; approval date: 26 May 2022), in accordance with the Declaration of Helsinki. The study was carried out in accordance with the STROBE Statement (Cuschieri, 2019). Informed consent was obtained from parents and athletes for minors, and from athletes of legal age before to the start of the study.

### Participants

The RStudio software (version 3.15.0, RStudio Inc., Boston, MA, USA) was used to calculate the sample size. The significance level was set a priori at α = 0.05, and the standard deviation was determined based on previous studies of the variable maturity offset (SD = 0.26) (Albaladejo-Saura et al., 2023). With an estimated error (d) of 0.07 years for the maturity offset variable, the minimum sample size of the present research was 47 athletes for a 95% confidence interval.

The sample was chosen through a non-probabilistic method based on coexistence. The group of selected participants was composed of athletes in the U-16, U-18, and U-20 categories who participated in the FAMU technification programme. On the other hand, the group of non-selected athletes was composed of athletes in the same categories who were not in the FAMU sports technification programme, but who were competing for club teams in the National League category.

**Figure 1:**
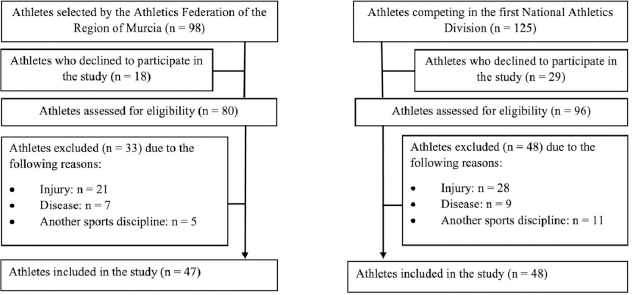
Sample selection flow chart.

A total of 95 athletes participated in the present research, of whom 46 were male (mean age = 18.31 ± 2.31 years) and 49 were female (mean age = 17.27 ± 1.44 years). The inclusion criteria for both groups were: (a) being federated in athletics, (b) belonging to the U-16, U-18 or U-20 categories; and (c) be specialised in short-distance running, jumping, throwing or combined track and field events. The exclusion criteria for the study were: (a) having suffered an injury or illness that prevented regular training or competition in the last three months, (b) failing to complete any of the tests outlined in the protocol, (c) having skipped over 20% of the training sessions in the preceding month, and (e) be federated in another sport. The flow chart with the sampling protocol can be found in Figure 1.

### Measures

#### Questionnaire Measurements

To collect information about socio-demographic aspects and athletic practice and training, participants answered an ad hoc questionnaire. They were asked about their age and self-identified sex, years of experience as federated athletes, days and hours of training per week, injuries or illnesses in the previous three months, practice of other sports as a federated athlete, and attendance at training sessions in the previous month.

The KIDMED questionnaire was used to assess adherence to the Mediterranean diet (AMD) (Serra-Majem et al., 2004). This questionnaire has been validated for assessing AMD in adolescents (Serra-Majem et al., 2004). It is composed of 16 items, with a dichotomous response (“yes” or “no”), and the scores range from 0 to 1 point for items positively related to AMD, and from −1 to 0 points for items negatively related to AMD (Serra-Majem et al., 2004). The final score that could be obtained ranged from 0 to 12 points (Serra-Majem et al., 2004).

The Questionnaire of Psychological Characteristics Related to Sport Performance (CPRD) was used to measure the psychological state (Gimeno et al., 2001). This questionnaire divides the mental state into five sub-scales: Motivation (M), Stress Control (SC), Team Cohesion (TCOH), Influence of Performance Evaluation (IPE), and Mental Skills (MSK). It is composed of a total of 55 items scored with a 5-option Likert scale (from “strongly agree” to “strongly disagree”). Previous studies have shown its optimal internal consistency for the total scale (α = 0.85), and for most of the subscales (αSC = 0.88; αIPE = 0.72; αM = 0.67; αTCOH = 0.78; αMSK = 0.34) (Olmedilla et al., 2019).

#### Anthropometric Measurements

Athletes underwent anthropometric assessment based on the anthropometric procedures from the International Society for the Advancement of Kinanthropometry (ISAK) (Esparza-Ros et al., 2019). Two level 3 and 4 anthropometrists with current ISAK accreditation took all the measurements. A SECA 862 scale with accuracy of 100 g (SECA, Hamburg, Germany) was used to measure body mass; a SECA 213 stadiometer with accuracy of 1 mm (SECA, Hamburg, Germany) was used to determine height and sitting height; a Harpenden calliper with accuracy of 0.2 mm (Harpenden, Harpenden, UK) was used to measure tricep, thigh, and leg skinfolds; a Lufkin tape with accuracy of 1 mm (Lufkin, Missouri City, TX, USA) was used to measure relaxed arm, mid-thigh and leg girths; and a Holtain small sliding calliper with accuracy of 1 mm (Holtain, Crymych, UK) was used to measure humerus, bi-styloid, and femur breadths.

All variables were measured in duplicate. If there was a difference between them of less than 5% in the skinfolds and 1% in the rest of the measurements, the final value of the variable was calculated as the mean of both values. However, if the margin of error exceeded these percentages, a third measurement was made, calculating the median to obtain the final value of the variable (Esparza-Ros et al., 2019).

The intra-evaluator technical error of measurement (TEM) was 0.03% for basic measurements, 2.24% for skinfolds, 0.36% for girths, and 0.48% for diameters. The inter-rater TEM was 0.05% for basic measurements and 2.83% for skinfolds, 0.52% for girths, and 0.75% for breadths. The correlation coefficient with a level-4 expert anthropometrist was 0.99 for basic measurements, 0.91 for skinfolds, 0.98 for girths and 0.96 for breadths. The room temperature was maintained constant at 24°C, and all measurements were conducted between 9:00 AM and 2:00 PM.

With the data obtained from the measurements, the body mass index (BMI) (Esparza-Ros and Vaquero-Cristóbal, 2023), Σ3-skinfold (Esparza-Ros and Vaquero-Cristóbal, 2023), fat (Slaughter et al., 1988), muscle (Poortmans et al., 2005), and bone mass in kg and percentage (Matiegka, 1921), corrected arm, thigh and leg girths (Esparza-Ros and Vaquero-Cristóbal, 2023), and corrected Σ3 girths (Esparza-Ros and Vaquero-Cristóbal, 2023) were calculated.

#### Maturation Assessment

The sex-specific formula (Mirwald et al., 2002) was used to estimate the age at which Age at Peak Height Velocity (APHV) was reached, as a measure of offset maturity. This method has been demonstrated to be accurate for estimating maturational status when compared to the left wrist radiograph, which is the gold standard method, with R2 = 0.92–0.89 for males and R2 = 0.91–0.88 for females (Mirwald et al., 2002). Furthermore, previous studies have demonstrated a coefficient of variance percentage (CV% = 0.8) and a low standard error (TE = 0.1) with this formula in adolescents (Towlson et al., 2017). The result of the equation is given in years since APHV if the value is positive, and in years until APHV if the value is negative (Mirwald et al., 2002).

#### Physical Fitness Tests

The assessment and familiarisation of the fitness tests were conducted under the supervision of four researchers with previous experience in this field. Each researcher supervised the same fitness tests throughout all measurements to avoid inter-rater TEM.

An ACUFLEX TESTER III box (Novel Products, Pittsburgh, PA, USA) was used to measure the sit-and-reach test. The distance attained was measured in cm. The test was conducted in a single attempt (Albaladejo-Saura et al., 2023), following the protocol of previous studies (Albaladejo-Saura et al., 2023; Vélez-Alcázar et al., 2024).

A force plate with a sampling frequency of 200 Hz (MuscleLab, Stathelle, Norway) was used to record the maximum countermovement vertical jump (CMJ) and squat jump (SJ) tests. For the CMJ test, athletes performed a maximal vertical jump with a countermovement (Dobbs et al., 2015). For the SJ assessment, the athlete performed a maximal vertical jump, without a countermovement, starting from a semi-squat position (Dobbs et al., 2015). The horizontal jump test was based on the execution of a maximum forward jump with both feet (Dobbs et al., 2015). The jump test protocols were the same as in previous studies (Vélez-Alcázar et al., 2024). In all tests, the result in cm was recorded (Mateo-Orcajada et al., 2023; Vélez-Alcázar et al., 2024).

A Takei Tkk5401 digital dynamometer (Takei Scientific Instruments, Tokyo, Japan) was used to measure handgrip strength in kg. Athletes were asked to grip the dynamometer, following the protocol from previous studies (Vélez-Alcázar et al., 2024). The test was performed with both arms, recording the force used in Nw (Mateo-Orcajada et al., 2023).

A three-kilogram medicine ball (Technogym, Cesena, Italy) was used to perform the medicine ball throw test. The test was conducted as described in previous studies (Albaladejo-Saura et al., 2023; Vélez-Alcázar et al., 2024). The distance of each throw was recorded in m.

Two pairs of photoelectric cells (Microgate, Bolzano, Italy) were used to measure the 30-m sprint test performance. Athletes initiated the movement from a squat position (Romero-Franco et al., 2017), as in previous studies (Vélez-Alcázar et al., 2024). Time was recorded in s. The choice to measure sprinting over 30 m of the linear sprint with no change of direction was made due to the importance of adjusting the assessment of this capacity to the environment of the sport being assessed (Oliva-Lozano et al., 2023; Willberg et al., 2023). Wind speed was monitored by placing a Gill-compact anemometer (Gill Athletics, Champaign, IL, USA) at mid-course, recording trials below 2 m per second in the running direction (World Athletics, 2023).

For the CMJ, SJ, horizontal jump, handgrip, medicine ball throw and sprint tests, two non-consecutive attempts were performed, with the final value being the maximum one from both attempts (Mateo-Orcajada et al., 2023; Vélez-Alcázar et al., 2024).

The Y-Balance Test consisted of a three-axis (anterior, posterior lateral and posterior medial) version of the Star Excursion Balance test (Plisky et al., 2009). The validated Y-Balance Test Kit (Functional Movement Systems, Chatam, VA, USA) was used for data collection (Plisky et al., 2009). Athletes performed the test barefoot and on a single supporting leg. They were asked to reach the maximum distance with the opposite limb to the supporting limb. Athletes were instructed that the toes of the supporting foot had to be behind the line of the platform and the foot had to be fully supported (heel included) during the entire execution. In addition, the athlete had to place the hands on the hips during the test. Three non-consecutive attempts with each leg were recorded, with the final result being the attempt at each direction and for each leg in which the greatest distance was achieved, provided the attempt was valid (Plisky et al., 2009).

### Procedures

The tests were conducted from 9.00 to 14.00 at the athletics track in Cartagena (Spain) to minimize potential variables that could impact the results. Initially, all athletes completed the questionnaires independently. Next, the anthropometric variables were evaluated. Subsequently, the sit-and-reach test was conducted without any warm-up beforehand, to eliminate the influence of warming up on the test results (Díaz-Soler et al., 2015). Following this, a standardized warm-up was conducted, beginning with 5 min of continuous running followed by joint mobility exercises. Athletes were then instructed on the proper execution of the fitness tests, with a familiarisation session provided for tests requiring technical precision (i.e., CMJ, SJ, horizontal jump, medicine ball throw, and Y-balance test). During this session, athletes were not required to perform maximal efforts to minimize the onset of fatigue. After the familiarization and warm-up session, athletes engaged in five minutes of progressively controlled sprints (40 m at 50%, 70%, and 90% effort) (Romero-Franco et al., 2017). Afterwards, athletes performed the CMJ, SJ, horizontal jump, medicine ball throw, handgrip, 30-m sprint, and Y-Balance tests in randomized order. A two-minute recovery period was given between each test, and a five-minute rest interval was provided between different tests. The testing protocol adhered to the guidelines established by the National Strength and Conditioning Association (NSCA) (Sands et al., 2012).

### Statistical Analysis

Kolmogorov-Smirnov, Levene’s, and Mauchly’s tests were used to evaluate normality, homogeneity and sphericity, respectively. Since all the variables analysed presented normal distribution, parametric tests were applied. The mean and standard deviation were determined for all the variables analysed. A one-way ANCOVA was performed to compare the differences between the selected and non-selected groups in the variables evaluated, determining the effect of the sex covariate on the differences between groups. The effect size was calculated with partial eta squared (η2). A Student's *t* test was performed to analyse the differences between the selected and non-selected athletes in the indicated variables, dividing the sample based on sex. The effect size was calculated with the Cohen's *d* (Cohen, 2013). A value of *p* < 0.05 was established to determine statistical significance. The SPSS software was used to perform the statistical analysis (v.25.0; SPSS Inc., Chicago, IL, USA).

## Results

Table 1 shows the differences between the selected and non-selected athletes based on sports and nutritional habits, psychological characteristics, and the effects of sex on this interaction. Table 2 shows the differences between the selected and non-selected athletes based on physical condition, anthropometric and derived variables, and the effects of sex on this interaction. The selected athletes obtained significantly higher values in the athletic experience variables (*p* = 0.003), weekly training (*p* = 0.004), maturity offset (*p* < 0.001), body mass (*p* < 0.001), height (*p* = 0.023), muscle mass (*p* = 0.009) and bone mass in kg and percentage (*p* = 0.044 and 0.000), BMI (*p* = 0.003), corrected arm (*p* < 0.001), thigh (*p* < 0.001) and leg girths (*p* = 0.007), ∑ corrected girths (*p* < 0.001), handgrip right (*p* = 0.049), CMJ (*p* < 0.001), SJ (*p* < 0.001) and horizontal jump tests (*p* = 0.001), medicine ball throw (*p* = 0.001), the sit-and-reach test (*p* = 0.024), and the Y-Balance test with both right and left legs (*p* < 0.001 and *p* = 0.013, respectively).

**Table 1:** Differences in sociodemographic, training and psychological variables between selected and non-selected, as well as the effect of sex.

	Selected(n = 47)	Not selected(n = 48)	ANOVA				Group*Sex			
	Mean ± SD	Mean ± SD	*F*	*p*	Ƞ^2^_p_	ICC 95%	F	*p*	Ƞ^2^_p_	ICC 95%
Age (years)	17.80 ± 2.44	17.70 ± 1.34	0.72	0.397	0.008	−0.50;1.15	4.00	0.022	0.079	−0.45;1.15
Athletic experience (years)	7.35 ± 3.32	5.21 ± 3.51	9.45	0.003	0.091	0.75;3.55	4.72	0.011	0.092	0.75;3.55
Weekly athletics training (days)	4.98 ± 0.86	4.46 ± 0.85	8.88	0.004	0.086	0.20;0.90	6.20	0.003	0.117	0.20;0.90
Weekly athletics training (hours)	8.50 ± 3.70	8.63 ± 2.54	0.03	0.848	0.000	−1.45;1.15	2.74	0.070	0.056	−1.40;1.20
Weekly gym training (hours)	3.50 ± 2.62	4.15 ± 2.71	1.40	0.239	0.015	−1.70;0.50	1.46	0.238	0.030	−1.70;0.50
Weekly other hours training (hours)	0.90 ± 2.13	1.60 ± 2.08	2.70	0.103	0.028	−1.55;0.20	2.74	0.070	0.056	−1.55;0.20
AMD (score)	8.00 ± 2.55	7.54 ± 2.40	0.74	0.390	0.008	−0.65;1.40	0.41	0.665	0.009	−0.65;1.45
Stress control (centile)	51.74 ± 29.08	53.44 ± 27.23	0.08	0.768	0.001	−13.45;9.60	4.90	0.010	0.095	−12.45;9.70
Influence performance (centile)	69.40 ± 29.84	68.73 ± 25.64	0.01	0.912	0.000	−10.55;12.40	2.00	0.141	0.042	−10.10;12.45
Motivation (centile)	68.81 ± 24.81	76.80 ± 22.55	2.70	0.104	0.028	−18.10;1.30	1.35	0.264	0.028	−18.15;1.35
Mental ability (centile)	48.40 ± 25.65	49.70 ± 27.70	0.05	0.812	0.001	−13.01;8.55	0.04	0.955	0.001	−13.10;8.60
Team relationship (centile)	47.71 ± 22.60	48.05 ± 27.20	0.00	0.947	0.000	−10.25;10.35	1.11	0.333	0.024	−10.50;10.00

AMD: adherence to the Mediterranean diet

**Table 2:** Differences in anthropometric and derived variables, and physical condition between selected and non-selected, as well as the effect of sex.

	Selected(n = 47)	Not selected(n = 48)	ANOVA					Group*Sex			
	Mean ± SD	Mean ± SD	*F*	*p*	Ƞ^2^_p_	ICC 95%	F		*p*	Ƞ^2^_p_	ICC 95%
Maturity offset (years)	3.85 ± 1.36	2.20 ± 1.00	45.8	<0.001	0.328	1.20;2.15	23.13		0.000	0.332	1.20;2.15
Body mass (kg)	69.00 ± 16.00	59.03 ± 9.15	14.1	<0.001	0.131	4.60;15.20	15.44		0.000	0.249	5.20;15.15
Height (cm)	172.12 ± 9.40	167.74 ± 9.22	5.33	0.023	0.054	0.50;8.15	43.20		0.000	0.482	1.90;7.55
Fat mass (kg)	13.41 ± 4.29	12.38 ± 2.68	2.02	0.158	0.021	−0.40;2.55	4.51		0.013	0.089	−0.40;2.45
Muscle mass (kg)	42.30 ± 4.70	37.34 ± 11.84	7.16	0.009	0.071	2.65;6.25	3.54		0.033	0.071	3.00;6.20
Bone mass (kg)	10.71 ± 1.60	10.04 ± 1.61	4.20	0.044	0.043	−0.07;1.30	52.72		0.000	0.531	0.25;1.20
Fat mass (%)	19.60 ± 4.90	21.12 ± 3.93	2.90	0.093	0.030	−3.30;0.35	35.95		0.000	0.436	−3.00;−0.25
Muscle mass (%)	42.28 ± 4.70	173.95 ± 661.25	1.90	0.171	0.020	1.25;8.65	0.94		0.394	0.020	1.20;8.65
Bone mass (%)	15.80 ± 1.83	17.07 ± 1.60	13.17	<0.001	0.123	−2.00;−0.60	14.60		0.000	0.239	−1.90;−0.60
BMI (kg/m^2^)	23.20 ± 4.58	20.92 ± 2.40	9.41	0.003	0.091	0.80;3.80	4.66		0.012	0.091	0.80;3.80
∑ 3 Skinfolds (mm)	40.14 ± 28.58	38.78 ± 19.37	0.07	0.785	0.001	−8.30;11.70	17.72		0.000	0.276	−7.70;9.50
Corrected arm girth (cm)	25.42 ± 3.45	22.93 ± 2.91	14.57	<0.001	0.134	1.15;3.75	38.01		0.000	0.450	1.50;3.60
Corrected thigh girth (cm)	46.81 ± 4.60	43.23 ± 3.72	17.60	<0.001	0.158	1.85;5.30	29.54		0.000	0.388	2.25;5.20
Corrected leg girth (cm)	33.60 ± 3.00	21.81 ± 3.40	7.47	0.007	0.074	0.45;3.00	21.22		0.000	0.313	0.70;2.95
∑ Corrected girths (cm)	105.81 ± 10.20	97.97 ± 0.02	15.90	<0.001	0.145	3.75;11.65	37.70		<0.001	0.448	4.85;11.25
Handgrip Right (Nw)	36.65 ± 9.53	32.96 ± 8.50	4.00	0.049	0.041	−0.15;7.30	38.30		0.000	0.451	1.15;6.75
Handgrip Left (Nw)	34.80 ± 9.81	31.40 ± 8.80	3.12	0.081	0.032	−0.70;6.90	35.40		0.000	0.432	0.55;6.40
CMJ (cm)	36.93 ± 8.90	30.45 ± 6.20	17.20	<0.001	0.155	3.40;9.65	42.30		0.000	0.476	4.35;9.30
SJ (cm)	34.65 ± 7.26	28.28 ± 5.62	23.12	<0.001	0.197	4.20;9.45	47.00		0.000	0.503	4.60;8.80
Horizontal jump test (cm)	217.15 ± 34.59	195.23 ± 29.02	11.31	0.001	0.107	8.25;34.22	35.07		0.000	0.430	11.95;32.95
Medicine ball throw (cm)	7.26 ± 1.60	6.02 ± 1.82	12.38	0.001	0.116	0.50;1.90	38.40		0.000	0.452	0.70;1.80
30 m sprint (m/s)	4.60 ± 0.50	4.70 ± 0.30	1.67	0.198	0.018	−0.25;0.10	27.01		0.000	0.367	−0.25;0.02
Sit-and-reach test (cm)	23.96 ± 9.84	19.80 ± 8.97	5.24	0.024	0.053	0.80;8.10	7.72		0.002	0.126	0.77;7.80
Y-Balance test Right leg (cm)	288.73 ± 47.61	253.96 ± 38.23	15.57	<0.001	0.142	−5.10;34.70	46.70		0.000	0.501	−4.30;35.00
Y-Balance test Left leg (cm)	253.90 ± 20.13	243.92 ± 18.32	6.42	0.013	0.064	3.45;18.55	4.50		0.014	0.088	3.60;18.70

BMI: body mass index; CMJ: counter movement jump; SJ: squat jump

The sex covariate showed a significant effect on the age variables (*p* = 0.022), athletic experience (*p* = 0.011), weekly training (*p* = 0.003), stress control (*p* = 0.010), maturity offset (*p* < 0.001), body mass (*p* < 0.001), height (*p* < 0.001), fat mass in kg and percentage (*p* = 0.013 and *p* < 0.001, respectively), muscle mass in kg (*p* = 0.033), bone mass in kg and percentage (*p* < 0.001), BMI (*p* = 0.012), ∑3-skinfolds (*p* < 0.001), corrected arm (*p* < 0.001), thigh (*p* < 0.001), and leg girths (cm) (*p* < 0.001), ∑ corrected girths (*p* < 0.001), handgrip right and left (*p* < 0.001), CMJ (*p* < 0.001), SJ (*p* < 0.001) and horizontal jump tests (*p* < 0.001), the medicine ball throw (*p* < 0.001), 30-m sprint speed (*p* < 0.001), the sit-and-reach test (*p* = 0.002), and the Y-Balance test with both right and left legs (*p* < 0.001 and *p* = 0.014, respectively).

Table 3 shows the differences in the male sample between the selected and non-selected athletes. The selected athletes showed greater maturity offset (*p* < 0.001), body mass (*p* = 0.006), fat mass in percentage (*p* < 0.001), muscle mass in kg and percentage (*p* < 0.001 and *p* = 0.044, respectively), bone mass in percentage (*p* = 0.002), the BMI (*p* = 0.014), corrected arm (*p* = 0.001), thigh (*p* < 0.001), and leg girths (*p* = 0.039), ∑ corrected girths (*p* < 0.001), the CMJ (*p* < 0.001), the SJ (cm) (*p* < 0.001), the horizontal jump test (*p* = 0.003), the medicine ball throw (*p* = 0.032), and the 30-m sprint test (*p* = 0.004).

**Table 3:** Differences in sociodemographic, training, psychological, anthropometric and derived variables, and physical condition between selected and not selected for male athletes.

	Selected(n = 22)	Not selected(n = 24)	Student *t*-test			
	Mean ± SD	Mean ± SD	t	*p*	*d*	ICC95%
Age (year-old)	18.63 ± 2.95	18.00 ± 1.60	−0.90	0.374	2.33	−2.00:0.80
Athletic experience (years)	7.30 ± 3.20	5.50 ± 3.90	−1.63	0.110	3.60	−3.86;0.40
Weekly athletics training (days)	5.20 ± 0.80	4.70 ± 1.00	−2.00	0.056	0.90	−1.03;0.01
Weekly athletics training (hours)	9.40 ± 4.30	9.30 ± 3.00	−0.10	0.917	3.70	−2.30;2.07
Weekly gym training (hours)	3.90 ± 3.80	4.60 ± 2.30	0.72	0.470	3.20	−1.20;2.60
Weekly other hours training (hours)	1.40 ± 2.90	2.00 ± 2.50	0.70	0.500	2.70	−1.20;2.15
AMD (score)	7.40 ± 2.30	8.20 ± 2.65	1.10	0.275	2.46	−0.66;2.30
Stress control (centile)	63.20 ± 24.10	59.60 ± 24.55	−0.50	0.620	24.32	−18.06;10.86
Influence performance (centile)	75.00 ± 29.00	75.66 ± 21.70	0.09	0.922	25.35	−14.50;16.00
Motivation (centile)	72.40 ± 25.80	73.50 ± 23.70	0.14	0.886	24.71	−13.65;15.75
Mental ability (centile)	52.30 ± 20.50	45.30 ± 26.10	−1.00	0.323	23.60	−21.00;7.07
Team relationship (centile)	44.80 ± 25.90	43.90 ± 27.80	−0.11	0.911	26.90	−17.10;15.30
Maturity offset (years)	3.80 ± 1.65	2.05 ± 1.20	−4.15	<0.001	1.45	−2.65;−0.90
Body mass (kg)	74.80 ± 18.20	63.10 ± 7.80	−2.85	0.006	13.80	−19.80;−3.42
Height (cm)	177.80 ± 8.40	174.80 ± 6.50	−1.35	0.184	7.45	−7.40;1.45
Fat mass (kg)	12.10 ± 4.00	11.82 ± 1.80	−0.30	0.759	3.00	−2.04;1.50
Muscle mass (kg)	31.40 ± 5.50	26.00 ± 3.70	−4.00	<0.001	4.65	−8.20;−2.70
Bone mass (kg)	11.80 ± 1.40	11.30 ± 1.20	−1.50	0.151	1.30	−1.30;0.20
Fat mass (%)	16.10 ± 2.50	18.80 ± 2.25	3.93	<0.001	2.35	1.35;4.15
Muscle mass (%)	42.70 ± 4.40	37.12 ± 11.80	−2.07	0.044	9.05	−10.95;−0.15
Bone mass (%)	16.20 ± 2.15	17.92 ± 1.30	3.30	0.002	1.75	0.66;2.75
BMI (kg/m^2^)	23.60 ± 5.30	20.62 ± 2.10	−2.60	0.014	3.95	−5.32;−0.65
∑ 3 Skinfolds (mm)	25.75 ± 22.65	27.30 ± 10.90	0.30	0.765	17.50	−8.85;12.00
Corrected arm girth (cm)	27.55 ± 3.10	24.70 ± 2.55	−3.45	0.001	2.80	−4.55;−1.20
Corrected thigh girth (cm)	49.70 ± 4.20	44.85 ± 3.40	−4.35	<0.001	3.80	−7.10;−2.60
Corrected leg girth (cm)	35.30 ± 2.50	33.35 ± 3.70	−2.15	0.039	3.20	−3.90;−0.10
∑ Corrected girths (cm)	112.55 ± 8.85	102.85 ± 8.00	−3.90	<0.001	8.40	−14.70;−4.70
Handgrip Right (Nw)	43.15 ± 9.30	38.45 ± 7.70	−1.90	0.068	8.50	−9.75;0.40
Handgrip Left (Nw)	40.65 ± 10.30	37.30 ± 7.80	−1.25	0.220	9.10	−8.75;2.10
CMJ (cm)	43.70 ± 7.00	33.70 ± 5.65	−5.35	<0.001	6.35	−13.75;−6.20
SJ (cm)	40.00 ± 5.45	31.45 ± 5.00	−5.55	<0.001	5.20	−11.55;−5.40
Horizontal jump test (cm)	239.50 ± 32.50	211.35 ± 27.30	−3.20	0.003	29.9	−45.95;−10.40
Medicine ball throw (cm)	8.25 ± 1.50	7.15 ± 1.85	−2.20	0.032	1.70	−2.10;−0.10
30 m sprint (m/s)	4.30 ± 0.30	4.50 ± 0.25	3.00	0.004	0.30	0.08;0.40
Sit-and-reach test (cm)	20.90 ± 11.10	18.15 ± 7.50	−1.00	0.321	9.40	−8.40;2.80
Y-Balance test Right leg (cm)	270.90 ± 93.80	248.75 ± 24.20	−1.15	0.270	67.10	−62.05;17.80
Y-Balance test Left leg (cm)	256.45 ± 23.75	245.85 ± 19.50	−1.70	0.100	21.65	−23.60;2.15

AMD: adherence to the Mediterranean diet; BMI: body mass index; CMJ: counter movement jump; SJ: squat jump

Table 4 shows the differences in the female sample between the selected and non-selected athletes. The selected athletes had more athletic experience (*p* = 0.011), weekly training (*p* = 0.025), AMD (*p* = 0.033), motivation (*p* = 0.023), maturity offset (*p* < 0.001), body mass (*p* = 0.006), height (*p* = 0.001), muscle mass in kg (*p* < 0.001), bone mass in kg (*p* = 0.003), corrected arm (*p* = 0.002), thigh (*p* = 0.009), and leg girths (*p* = 0.018), ∑ corrected girths (*p* = 0.003), handgrip right and left (*p* = 0.029 and *p* = 0.014, respectively), the CMJ (*p* = 0.016), the SJ (*p* < 0.001), the horizontal jump test (*p* = 0.007), the medicine ball throw (*p* < 0.001), the sit-and-reach test (*p* = 0.015), and the Y-Balance test with the left leg (*p* = 0.011).

**Table 4:** Differences in sociodemographic, training, psychological, anthropometric and derived variables, and physical condition between selected and not selected for female athletes.

	Selected(n = 25)	Not selected(n = 24)	Student’s *t* test			
	Mean ± SD	Mean ± SD	t	*p*	*d*	ICC95%
Age (years)	17.35 ± 1.85	17.20 ± 0.95	−0.30	0.775	1.45	−0.95;0.71
Athletic experience (years)	7.50 ± 3.60	5.00 ± 3.20	−2.65	0.011	3.35	−4.45;−0.60
Weekly athletics training (days)	4.90 ± 1.00	4.30 ± 0.70	−2.35	0.025	0.83	−1.05;−0.07
Weekly athletics training (hours)	7.70 ± 3.00	8.00 ± 1.90	0.44	0.660	2.55	−1.15;1.80
Weekly gym training (hours)	3.30 ± 1.80	3.80 ± 2.50	0.83	0.415	2.20	−0.73;1.75
Weekly other hours training (hours)	0.20 ± 1.25	1.30 ± 1.70	1.90	0.067	1.44	−0.05:1.60
AMD (score)	8.45 ± 2.75	6.95 ± 2.00	−2.20	0.033	2.45	−2.90;−0.13
Stress control (centile)	41.30 ± 30.15	47.30 ± 28.90	0.70	0.477	29.5	−10.92;23.03
Influence performance (centile)	65.06 ± 31.15	62.10 ± 27.80	−0.35	0.728	29.5	−19.95;14.03
Motivation (centile)	64.82 ± 24.10	80.10 ± 21.35	2.35	0.023	22.7	2.20;28.40
Mental ability (centile)	43.20 ± 28.25	54.06 ± 29.12	1.35	0.191	28.6	−5.62;27.35
Team relationship (centile)	59.90 ± 19.80	52.25 ± 26.50	0.20	0.840	23.3	−12.05;14.75
Maturity offset (years)	3.90 ± 1.15	2.35 ± 0.70	−5.75	<0.001	0.95	−2.10;−1.00
Body mass (kg)	63.80 ± 12.20	54.95 ± 8.80	−2.90	0.006	10.6	−15.00;−2.72
Height (cm)	167.03 ± 7.40	160.70 ± 5.35	−3.45	0.001	6.44	−10.05;−2.65
Fat mass (kg)	14.70 ± 4.40	12.95 ± 3.35	−1.55	0.128	3.90	−4.00;0.51
Muscle mass (kg)	26.35 ± 3.30	22.60 ± 2.85	−4.30	<0.001	3.00	−5.55;−2.00
Bone mass (kg)	9.70 ± 1.10	8.85 ± 0.90	−3.10	0.003	1.00	−1.45;−0.30
Fat mass (%)	22.90 ± 4.35	23.45 ± 4.00	0.55	0.608	4.14	−1.77;3.00
Muscle mass (%)	42.00 ± 5.10	37.60 ± 12.15	−1.65	0.105	9.24	−9.67;0.95
Bone mass (%)	15.45 ± 1.50	16.22 ± 1.40	1.90	0.064	1.45	−0.04;1.60
BMI (kg/m^2^)	22.85 ± 4.05	21.25 ± 2.70	−1.65	0.107	3.45	−3.60;0.36
∑ 3 Skinfolds (mm)	53.50 ± 27.70	50.25 ± 19.40	−0.50	0.642	24.0	−17.00;10.60
Corrected arm girth (cm)	23.45 ± 2.50	21.20 ± 2.15	−3.40	0.002	2.35	−3.60;−0.90
Corrected thigh girth (cm)	44.20 ± 3.35	41.60 ± 3.40	−2.75	0.009	3.35	−4.55;−0.70
Corrected leg girth (cm)	32.00 ± 2.50	30.30 ± 2.25	−2.45	0.018	2.35	−3.00;−0.30
∑ Corrected girths (cm)	99.60 ± 7.20	93.10 ± 7.30	−3.15	0.003	7.25	−10.70;−2.35
Handgrip Right (Nw)	30.70 ± 5.03	27.50 ± 5.01	−2.25	0.029	5.02	−6.15;−0.35
Handgrip Left (Nw)	29.10 ± 4.90	25.50 ± 4.90	−2.55	0.014	4.90	−6.40;−0.80
CMJ (cm)	31.10 ± 5.90	27.20 ± 4.95	−2.50	0.016	5.45	−7.00;−0.75
SJ (cm)	30.10 ± 5.50	25.10 ± 4.35	−3.55	<0.001	4.95	−7.85;−2.15
Horizontal jump test (cm)	196.20 ± 21.35	179.15 ± 20.90	−2.85	0.007	21.1	−29.20;−4.90
Medicine ball throw (cm)	6.30 ± 1.00	4.95 ± 0.95	−4.95	<0.001	1.00	−1.95;−0.80
30 m sprint (m/s)	4.90 ± 0.45	4.85 ± 0.30	−0.15	0.900	0.35	−0.25;0.20
Sit-and-reach test (cm)	27.15 ± 7.60	21.40 ± 8.25	−2.55	0.015	7.95	−10.25;−1.15
Y-Balance test Right leg (cm)	246.55 ± 19.20	237.55 ± 15.90	−1.80	0.085	17.7	−19.15;1.15
Y-Balance test Left leg (cm)	252.40 ± 16.25	240.90 ± 14.14	−2.65	0.011	15.3	−20.30; −2.70

AMD: adherence to the Mediterranean diet; BMI: body mass index; CMJ: counter movement jump; SJ: squat jump

## Discussion

The main objective of this research was to analyse the differences between selected and non-selected athletes taking part in technical training programs, considering sociodemographic, training, psychological, anthropometric variables, derived variables and physical condition, of athletes in training and in relation to sex. In the general sample, the selected athletes were found to have more experience in athletics and to train more days per week. Previous studies carried out with Olympic athletes from various sport disciplines have shown the importance of having more sports experience and training volume to be able to compete at the highest level (Issurin, 2017), and that the most successful athletes tend to have a greater training volume (Issurin, 2015). However, when the sample was divided according to sex, females showed the same differences, while no differences in males were observed in variables related to training volume or athletics experience. This could be due to the fact that only high-performance athletes participated in the present research, thus even the group of non-selected male athletes consisted of athletes with a long sporting career and a high volume of training. On the other hand, considering females, previous studies have pointed out that as there are fewer females athletes (Gobierno de España, Ministerio de Cultura y Deporte, 2023), there may be less competition to reach the highest level, which could result in athletes with a less extensive sporting career than in males (Leiva-Arcas et al., 2021). Therefore, in light of the present research, with respect to the training categories, experience and volume of training could be a determining variable in the possibility of being selected for females, but not for males.

Another notable result of the present investigation was that the selected athletes had a more advanced state of maturation than the non-selected ones, both in the general sample and when divided by sex. Previous studies have indicated that young athletes who mature earlier than their peers are more likely to be selected for high-performance programs (Albaladejo-Saura et al., 2023). This may be due to the fact that the physical and physiological changes experienced during maturation have an effect on sports performance, generating a competitive advantage for athletes who mature earlier (Malina and Bouchard, 1991). Furthermore, this phenomenon occurs regardless of the sex of the athlete (Malina and Bouchard, 1991). It is important to highlight that the competitive advantage derived from early maturation during the formative years disappears once the growth stage ends (Gonçalves et al., 2012). Therefore, a higher performance at these ages due to early maturation does not guarantee continued performance in future stages (Albaladejo-Saura et al., 2021; Gonçalves et al., 2012). Given the notable disparity in the state of maturation between the selected and non-selected athletes found in the present research, irrespective of the sex of athletes, sports training programs should consider this aspect when establishing the selection criteria for these programs both in males and females, to decrease the appearance of the phenomenon of “waste of talent” (Pearson et al., 2006).

In the present study, it was found that the selected athletes had greater muscle mass, corrected arm, thigh and leg girths, and ∑ corrected girths; differences were also found when dividing the sample based on sex in both males and females. Previous studies have shown that muscle mass positively influences power production, establishing a synergistic relationship between increased muscle mass and power production (Kraemer and Newton, 2000). This ability is crucial for sports performance in most sports modalities and tests (Kraemer and Newton, 2000). The greater muscle development observed in the selected athletes, both in general and in the extremities, could be attributed to different factors. The main factor that contributes to a greater muscle development in the selected athletes is their more advanced state of maturation. Previous research suggests that muscle development may be related to biological maturation, with early maturing individuals showing greater muscle development both in males and females (Albaladejo-Saura et al., 2021). This could be because the growth of muscle mass during puberty is linked to an increase in circulating testosterone that is produced around the APHV in both sexes, although the increase is much more exponential in males (Handelsman et al., 2018). Secondly, considering females, the selected athletes trained more. This increase in training could provide an additional stimulus for the development of muscle mass, especially once the APHV has passed (Handelsman et al., 2018), as is the case of the female athletes in the present investigation. Therefore, variables related to muscle mass could be determinant in the chances of athletes in training to be selected for technification programmes, both in males and females, although the influence of maturation on muscle mass at this stage of growth means that it is not a variable that can predict long-term performance.

Furthermore, in the present study, it was observed that the selected athletes had a higher bone mass, in the general sample, and in both males and females, and height in the general sample as well as in the sample of females. The increase in bone development during puberty is related to the growth hormone (GH), of which levels increase exponentially around APHV in both males and females (Saenger, 2003). Therefore, it is likely that athletes who are more advanced in their maturation, such as those in the group selected for this study, have greater heights and bone masses (Albaladejo-Saura et al., 2021; Malina and Bouchard, 1991). It is also important to highlight the influence of increased muscle mass on the development of bone mass, as it has been observed that athletes with a greater muscle mass also have a greater bone mass (Greene et al., 2012). This could be because bone mass is a living tissue that responds to the load generated by muscle traction and impact, resulting in increased bone mineral density (Greene et al., 2012). On the other hand, the lack of size differences in males could be due to the fact that the sample of non-selected athletes was also composed of high-performance athletes. Given that height is one of the anthropometric variables most closely related to sports performance (Khosla and McBroom, 1985; Stanula et al., 2013), it is possible that it had a high specific weight in the selection of athletes that made up this group of high-performance athletes. Whereas in females, as there is less competition to reach the elite (Leiva-Arcas et al., 2021), it is possible that this variable is not a defining one for belonging to a high-performance group, although it is defining for being selected, in light of the results of the current study. Although these results are promising, some questions remain. In general terms, due to the influence of maturation on bone mass and height, their use in sports talent detection programmes during the growth stage should be carried out with caution.

However, it was found that there were no differences in most of the variables related to adiposity, neither for the general sample nor considered by sex. In males, previous studies have suggested that fat mass does not change significantly during growth due to maturation (Albaladejo-Saura et al., 2021), and that changes in this tissue depend more on energy balance (Aerenhouts et al., 2011). In this sense, in the present investigation, similar AMD scores were found between both groups in males, which could reflect very similar nutritional habits (Mateo-Orcajada et al., 2023). Previous research has also shown that in athletes belonging to the same competitive category, small differences in training volume seem not to be sufficient to affect energy balance and to generate changes in adipose tissue (Vaquero-Cristóbal et al., 2019). Therefore, in light of the present research, nutritional planning needs to be carried out to change the adiposity of male athletes in training (Vaquero-Cristóbal et al., 2019). For females, previous studies have suggested that early maturers may have a greater fat development than late maturers (Albaladejo-Saura et al., 2021, 2022). However, in the present investigation, the selected female athletes did not have a higher fat mass than their non-selected peers, even though they were more mature. This could be because the selected female athletes also showed a higher AMD and a higher training volume, which could condition them to not to have a positive energy balance, thus avoiding an increase in fat mass in the selected group despite their more advanced maturation (Vaquero-Cristóbal et al., 2019). Given these promising results, the differences in adiposity variables in athletes in training, between different categories and according to their sporting level, need further investigation.

In the present investigation, it was observed that the group of selected athletes had a higher body mass and a higher BMI than the group of non-selected athletes; these differences were found both in the general group and in the group of males. With regard to females, those selected had a greater body mass than those who were not selected. However, the body mass variable does not allow us to differentiate between the particular components of body composition (Esparza-Ros and Vaquero-Cristóbal, 2023), thus changes in it or in the BMI have been described as non-specific indicators in the population of athletes (Albaladejo-Saura et al., 2023). Not in vain, the differences between groups in these variables shown in the present research could be due to differences in muscle mass depending on the maturation state (Albaladejo-Saura et al., 2021). Furthermore, the same differences were found in body mass and the BMI between the selected and non-selected male groups, due to the homogeneity of the height variable shown by both groups. Therefore, in this population, the differences in the BMI are conditioned by changes in body mass (Esparza-Ros and Vaquero-Cristóbal, 2023). In contrast, in females, as there were significant differences between the selected and non-selected groups in both body mass and height, no significant differences in the BMI were found (Esparza-Ros and Vaquero-Cristóbal, 2023). Based on the above, differences in body mass and the BMI should be used with caution in the selection or screening process for sporting talent.

An important finding of the study was that the selected athletes showed significantly higher values in vertical jump tests (CMJ and SJ), the horizontal jump, the medicine ball throw, the handgrip test and the sprint test, both in the general sample and in relation to sex. Within athletics, several disciplines involve explosive movements, the performance of which can benefit from limb strength and power (Dobbs et al., 2015). A possible cause for the differences found in these physical fitness tests could be that strength and power are related to higher values of muscle mass (Kraemer and Newton, 2000). Therefore, the differences in muscle mass observed between the selected and non-selected groups could partly explain the differences in performance on these tests. Another possible explanation would be that training creates neuromuscular adaptations in inter- and intramuscular coordination (McQuilliam et al., 2020), which could favour performance in these tests (Albaladejo-Saura et al., 2023). As the group of selected athletes had been training for a longer period of time, it is possible that the adaptations they had in this sense were somewhat greater than the components of the not selected group.

In the sit-and-reach and Y-Balance tests, athletes in the selected group showed higher scores than those who were not selected, with differences found in the general sample and the group of females. Hamstring extensibility tends to shorten with age (Díaz-Soler et al., 2015), with maturation not affecting this ability (Albaladejo-Saura et al., 2021). Numerous studies have systematically analysed the effect of sports practice on this ability, finding a direct relationship between stretch volume and hamstring extensibility (Muyor et al., 2014). Therefore, changes found in females in the selected group could be a result of their greater volume of stretching due to having a greater volume of weekly training. Regarding the Y-balance test, previous studies have indicated its performance could depend on balance. This component is frequently introduced in athletics training, given its influence on power and motor control in the execution of jumps and single-leg landings, fundamental in athletic events (Chelly et al., 2015). Therefore, the results found in females in the selected group could be the consequence of a greater volume of plyometric and strength training in their lower extremities. In the male group, there was no difference in the weekly training volume, which may explain why there was no difference in the stretching exercise volume between the two groups, and thus in the sit-and-reach test results. In addition, there were no differences in the volume of balance training, thus no differences in the Y-balance test score were found in males depending on the group.

Among the strengths of this research, it must be underlined that this is the first study that characterized variables differentiating between selected and non-selected athletes from athletics specialization training programs. The main practical implications of this research are that in the future, the modulating effect of biological maturation and the volume of training and sports experience should be considered when selecting athletes for different sports technicalization programs. Coaches, scouts, clubs, and sports organizations must consider that by discarding athletes in early stages for these reasons, they could create a “waste of talent” at an early age.

Despite the above, the present study is not without limitations. Among them is the sample size, which prevents differentiating between the different disciplines of athletics when carrying out the analysis in this study. Furthermore, the cross-sectional design limits the ability to establish causal relationships between the variables analysed. The use of maturation estimation equations instead of wrist and hand radiographs, considered the gold standard (Malina and Bouchard, 1991), is also a limitation. All of these issues need to be addressed in the future.

## Conclusions

In conclusion, the present investigation found that athletes who participated in sports training programs had a more advanced maturational state, a greater muscle and bone development, and a greater performance in physical fitness tests that depended on strength, power, speed, flexibility, and stability. In addition, in females, a greater sports experience and training volume, a higher AMD, and a higher performance in the flexibility and balance tests were also found.

Therefore, coaches, scouts, clubs, and sports bodies in charge of the development of young athletes must be aware of the modulating effect of biological maturation, the volume of training, and sports experience, when selecting athletes for the different sports technicalization programs, in order to avoid the exclusion of athletes at an early age, who could have a high level when the growth stage ends. In this way, the detection of sporting talent should be avoided that is based exclusively on variables of muscle development, bone development, physical performance in jumping, sprinting or explosive strength at this age of growth, which are variables directly related to maturation, giving an advantage to early maturers, which is then equalised when the maturation process is complete. Nor should they be based exclusively on variables such as ranking, which are highly conditioned by the above.

Finally, any sports talent detection programme that seeks long-term and not exclusively immediate performance must consider whether the factors that make an athlete stand out are not exclusively that he or she has matured earlier has trained more intensively or for a longer period of time.
